# Antibacterial effect of curcumin on *Salmonella* Typhimurium: *In vitro* and food model studies

**DOI:** 10.17221/114/2023-VETMED

**Published:** 2024-04-26

**Authors:** Goknur Terzi Gulel, Sibel Kanat, Esra Kucukgoz

**Affiliations:** Department of Food Hygiene and Technology, Faculty of Veterinary Medicine, Ondokuz Mayis University, Samsun, Turkiye

**Keywords:** chicken, curcumin, *S.* Typhimurium

## Abstract

Salmonellosis is a major foodborne disease transmitted from contaminated poultry products worldwide. Although a wide variety of chemical agents are used in the prevention of foodborne *Salmonella* spp. infections, consumers prefer natural additives, that do not harm human health and do not impair the characteristics of food. Curcumin is a yellow-coloured, hydrophobic polyphenol obtained from the rhizome of the *Curcuma longa* L. plant known as turmeric. The purpose of this study was to evaluate curcumin’s antibacterial activity against *S*. Typhimurium in chicken meat and *in vitro*. In the first step, chicken samples were experimentally contaminated with *S.* Typhimurium at a level of 2.8 × 10^–7^ CFU/ml. Then, they were kept in a 1, 2, and 3% curcumin solution for 15 minutes. At the end of the treatment, chicken samples were stored at +4 °C. The number of *S*. Typhimurium in chicken samples was determined according to EN ISO 6579-1. In the result of the study, the number of *S.* Typhimurium decreased by 2.37, 2.71, and 2.84 log levels at the end of the 6^th^ day as a result of the 1, 2 and 3% curcumin treatment, respectively. The MIC value of curcumin was determined to be 362 μg/ml for *S.* Typhimurium.

*Salmonella* is a gram-negative foodborne pathogen that is a significant risk for global public health (Jajere 2019). Consuming foods contaminated with *Salmonella* species causes symptoms including headaches, fever, nausea, vomiting, myalgia, abdominal cramps, and diarrhoea (Punchihewage-Don et al. 2022). Raw or undercooked meat and meat products, poultry, eggs, and unpasteurised milk are major sources of infections (Ehuwa et al. 2021). It was estimated that, in the United States, 420 deaths, 1.35 million infections, and 26 500 hospitalisations occur annually due to the foodborne pathogen *Salmonella* (CDC 2023).

Turmeric (*Curcuma longa* L.) is a yellow-flowered, large-leafed, perennial plant species of the Zingiberaceae family that grows in tropical and subtropical regions.

Its homeland is South Asia, and it is widely used in China, India, and Southeast Asia as a food colouring, spice, and preservative. Due to its yellow colour, it is called “Indian Saffron” or “Golden Spice” (Goel et al. 2008).

There are three main curcuminoids in turmeric such as bisdemethoxy curcumin (10–15%), demethoxy curcumin (20–27%) and curcumin (60–70%) (Nelson et al. 2017).

Curcumin is a yellow-orange hydrophobic polyphenol obtained from the rhizome of the turmeric (*Curcuma longa* L.) plant. It is chemically referred to as 1,7-*bis*(4-hydroxy-3-methoxyphenyl)-1,6-heptadiene-3,5-dione (Tripathy et al. 2021). The European Union (EU) has approved curcumin as a food additive. It exists by several names, including E100, natural yellow 3, CI 75300, and diferuloylmethane (Sharifi-Rad et al. 2020). Curcumin is used as a food colourant in some foods such as meat, fish, dairy products, ice cream, eggs, beverages, cereal, mustard, pickles, and bakery products between doses of 50 and 500 mg/kg (EFSA 2014).

Curcumin has different effects, such as being an anti-carcinogenic, anti-inflammatory, antioxidant, and having a hypoglycaemic effect. Additionally, it has been used in the treatment of a variety of diseases, including diabetes, inflammatory bowel disease, arthritis, cancer, cystic fibrosis, multiple sclerosis, skin conditions, lung conditions, liver conditions, Alzheimer’s disease, and autoimmune diseases (Prasad et al. 2014).

The purpose of this research was to investigate the antibacterial effect of curcumin on *S.* Typhimurium *in* *vitro* and *in* *vivo* (in chicken samples) and to determine the effect of curcumin on the sensory characteristics of food.

## MATERIAL AND METHODS

### Sample collection and preparation of curcumin

Chicken meat samples were obtained from the local market in Samsun, Türkiye. Curcumin was purchased from Sigma (C 1386; St. Louis, MI, USA) and it was prepared in 1, 2, and 3% concentrations in 20% dimethyl sulfoxide (DMSO, Isolab 914.036; Wertheim, Germany). *S*. Typhimurium ATCC 13311 was purchased from Microbiologics Company (Saint Cloud, MN, USA).

### Determination of the *in vitro* antibacterial activity

The disc diffusion test was carried out following the methods of the Clinical and Laboratory Standards Institute for the determination of antibacterial activity (CLSI 2020). *S*. Typhimurium ATCC 13311 was grown in Mueller-Hinton broth (MHB) (Merck 1.10293) and incubated at 35 °C for 2–6 h until turbidity. The suspension was adjusted to 0.5 McFarland (1.5 × 10^–8^ CFU/ml) with a densitometer (DEN*-*1; Biosan, Riga, Latvia). Bacterial suspensions were diluted to a final concentration of 1 × 10^–6 ^CFU/ml. Then, 100 μl of the suspension was spread onto Mueller-Hinton agar (MHA, Merck 1.05437) plates uniformly. Discs with a diameter of 6 mm were placed on the Petri dishes. Then, 25 μl of curcumin prepared in dimethyl sulfoxide (DMSO) at different concentrations (from 200 mg to 12.5 mg) was added to the empty discs. Only DMSO (25 μl) was added to one of the discs. All the discs were incubated at 35 °C for 16–20 hours. A gentamicin disc (10 μl/ml) was used as a positive control (Gupta et al. 2015). The results were classified as follows based on the width of the inhibition zone: 9 mm indicated no activity; 9–12 mm indicated moderate activity; 13–18 mm indicated activity; and >18 mm indicated considerable activity (Alves et al. 2000). All the analyses were performed in triplicate.

### Determination of the minimum inhibitory concentration (MIC)

The minimum inhibitory concentration (MIC) value of the curcumin was defined by the microdilution method using twofold serial dilutions in a Mueller-Hinton broth medium according to the CLSI (2020). The bacterial inoculum was adjusted to the 0.5 McFarland (1.5 × 10^–8^ CFU/ml) standard and the final inoculum was diluted to 1 × 10^–5^ CFU/ml. The curcumin solution was double diluted to 2 900 μg/ml, 1 450 μg/ml, 725 μg/ml, 362 μg/ml, 181 μg/ml, 90 μg/ml and 45 μg/ml. The MIC value was determined to be the lowest amount of this compound which prevented any discernible microbiological growth (Gunes et al. 2016).

### Chicken meat preparation

The chicken meat samples were divided into four pieces (control, 1% curcumin, 2% curcumin, and 3% curcumin with %20 DMSO). Firstly, the samples were contaminated by dipping in tryptic soy broth (TSB) containing 2.8 × 10^–7^ CFU/ml *S*. Typhimurium ATCC 13311 and left at room temperature for 15 min to allow attachment. Then, the samples were removed from the bacteria tank, dipped in the 1, 2, and 3% curcumin solutions, and left for 15 minutes. The control group samples were dipped in the TSB containing 2.8 × 10^–7^ CFU/ml *S*. Typhimurium. Then, the control samples were dipped in the 20% DMSO (without curcumin). Up to the analysis, all the samples were kept at +4 °C.

### Microbiological analyses

The microbiological analyses of the samples were performed on days 0, 2, 4, and 6 of storage. The number of *S*. Typhimurium in the chicken samples was determined according to EN ISO 6579 (ISO 2017). Briefly, 25 g of chicken sample was taken and 225 ml of buffered peptone water (Merck 107228; Darmstadt, Germany) was added, and the sample was homogenised for 3 min with a stomacher (Interscience, St Nom La Breteche, France). Ten-fold serial dilutions were prepared with buffered peptone water and spread on XLT-4 agar (Merck 1.13919) then incubated at 37 °C for 24–48 hours. The bacterial count was determined by counting the black-centred colonies with smooth edges.

### Sensory evaluation

Chicken meat samples were prepared in approximately 100 g portions, and curcumin was added at 1, 2, and 3%. Curcumin was used directly in a powder form without being prepared in any solvent. Curcumin was not added to the control group. Then all the meat samples were cooked in a pan at a central temperature of 72** °**C. The samples were evaluated by ten panellists using a nine-point hedonic scale for their sensory evaluation (colour, odour, flavour, texture, appearance, and overall acceptability (Govaris et al. 2010).

### Statistical analysis

All the measurements were analysed in triplicate. A one-way analysis of variance (ANOVA) and Tukey’s Post Hoc Tests were used in the SPSS v15 statistical package programs to evaluate the data.

## RESULTS

### *In vitro* antimicrobial activity and MIC value

In this study, at the end of the disc diffusion test, the antibacterial activity was determined by measuring the inhibition zone diameters around the discs. The inhibition zone diameter was 13 mm for 200 mg/ml of curcumin, 12 mm for 100 mg/ml of curcumin, and 11 mm for 50 mg/ml of curcumin against *S*. Typhimurium ATCC 13311. The DMSO disc diameter was 9 < mm, The Gentamicin disc diameter was 22 mm. Accordingly, the active dose at which curcumin was effective was found to be 200 mg/ml. The microdilution test was used to determine the MIC value of the curcumin. The MIC value was found to be 362 μg/ml for *S.* Typhimurium.

### Microbiological analyses

The chicken meat was experimentally contaminated with *S.* Typhimurium at a level of 2.8 × 10^–7^ CFU/ml. The decrease in the number of *S.* Typhimurium as a result of the subsequent administration of 1, 2, and 3% curcumin is shown in Table 1. Accordingly, on the sixth day of storage, the number of *S.* Typhimurium in the control group did not change significantly, while the number of bacteria in the 1, 2, and 3% curcumin groups decreased by 2.37, 2.71, and 2.84 log/CFU, respectively. It was determined that as the dose of curcumin increased, its antibacterial activity increased.

**Table 1:** Log reduction in the *S*. Typhimurium counts on storage days 0, 2, 4, and 6

Initial *S*. Typhimurium counts (log)	Storage duration (day)	Reduction in *S*. Typhimurium count (log10)			
		control	1% curcumin	2% curcumin	3% curcumin
7.44	0	1.89 ± 0.01^a^	1.90 ± 0.01^a^	1.91 ± 0.01^a^	2.67 ± 0.01^b^
	2	1.89 ± 0.01^a^	2.10 ± 0.01^c^	2.54 ± 0.01^d^	2.73 ± 0.01^e^
	4	1.87 ± 0.01^a^	2.30 ± 0.01^f^	2.61 ± 0.01^g^	2.75 ± 0.01^e^
	6	1.88 ± 0.01^a^	2.37 ± 0.01^h^	2.71 ± 0.01^i^	2.84 ± 0.01^j^

^a–j^Between the mean values indicated by various letters, there is a statistically significant difference at *P *≤ 0.05

### Sensory evaluation

**Figure 1:**
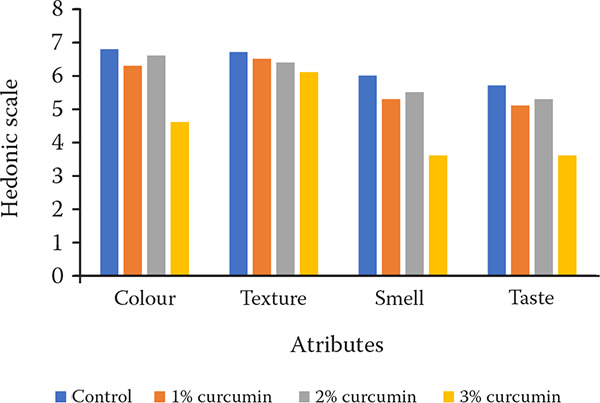
Sensory evaluation of curcumin in chicken meats

In the present study, the sensory changes in chicken meats that were treated with 1, 2, and 3 curcumins were evaluated in terms of the colour, taste, odour, structure, and appearance (Figure 1). Colour is an important parameter in food quality. Chicken meats treated with 1% and 2% curcumin were accepted by the panellists in terms of the colour. On the other hand, it was determined that the colour of the chicken meat treated with 3% curcumin was too yellow, and it was not accepted. According to the evaluation made in terms of the smell and taste, it was observed that 3% curcumin scored low because the meat was sharp-smelling and bitter-tasting as the dose of curcumin increased. No significant difference in texture was observed among the three groups. As a result, the chicken meat that was treated with 1% and 2% curcumin was accepted in terms of the colour, odour, taste, and appearance, while 3% curcumin was found to be unacceptable.

## DISCUSSION

### *In vitro* antimicrobial activity and MIC value

Salmonellosis is an important public healthcare issue causing foodborne infections (Dar et al. 2017). Various antibiotics have been used in the past to combat microorganisms. However, new natural agents are sought against antibiotic resistance caused by the unconscious and widespread use of antibiotics. Based on the understanding of the neurotoxic and carcinogenic effects of synthetic preservatives on human health, consumers want to consume natural, non-toxic, minimally processed foods (Lan et al. 2023). For this reason, in the present study, the antibacterial activity of curcumin, which is a natural antimicrobial agent obtained from the rhizome of the turmeric plant, on *S.* Typhimurium was investigated *in vitro* and *in vivo*. It has been reported in previous studies that the use of curcumin alone or in combination with other antibacterial agents caused strong antimicrobial activity (Teow et al. 2016).

In the present study, the MIC value of curcumin prepared with DMSO against *S.* Typhimurium ATCC 13311 was found to be 362 μg/ml with the microdilution test. It has been reported in previous studies that the MIC values of curcumin against gram-positive and gram-negative bacteria ranged between 31.25 μg/ml and 5 000 μg/ml (Tyagi et al. 2015; Adamczak et al. 2020). Altunatmaz et al. (2016) found the MIC value of curcumin prepared with ethanol against *S.* Typhimurium ATCC 14028E to be 250 μg/ml with the macrodilution method. Keyvan et al. (2022) found the MIC value of curcumin prepared in DMSO for *S.* Enteritidis as 132.5 μg/ml with the broth microdilution method. On the other hand, Lawhavinit et al. (2010) reported that ethanol turmeric extract, hexane turmeric extract, and curcuminoids (curcumin) did not show inhibitory effects against all *S.* Typhi, *S.* Typhimurium, and *S.* Enteritidis.

In studies conducted by different researchers, different results were found in the MIC values of curcumin. This difference might have occurred because 1) the type of curcumin, 2) the method of obtaining it, 3) the different solvents used in its preparation, 4) the sources of bacteria, and 5) the methodological differences used in the MIC tests. The microdilution test, which is used to determine the MIC value, is a widely used method. In the present day, many studies are needed to comprehensively investigate the antimicrobial effects of curcumin against different bacteria using a standard method (Teow et al. 2016; Dai et al. 2022).

Studies have reported that the antibacterial activity of curcumin varies depending on the solvents used (e.g., DMSO, ethanol, methanol, hexane, and water). In this case, the question arises whether the antibacterial activity of curcumin originates from these solvents or itself. In the present study, as a result of the disc diffusion test, it was found that curcumin was effective at moderate and active levels with an inhibition zone diameter of 11–13 mm against *S.* Typhimurium ATCC 13311. The fact that the zone diameter was below 9 mm in the discs where only DMSO was used showed that the effectiveness of the DMSO only was not very high.

Gul and Bakht (2015) reported that 12 μg of turmeric methanol extract (at 121 °C) had a 10 mm inhibition zone against *S*. Typhi and an *n*-hexane and chloroform extract had a 10–10.5 mm inhibition zone and a water extract (at 121 °C) had a 0 mm inhibition zone. Researchers have stated that samples of turmeric extracted with methanol, chloroform, and *n*-hexane had activity against *Salmonella*. However, they have reported that the water extract was not effective. Also, Sakha et al. (2018) reported that 200 mg/ml of an ethanolic extract of *C.* *longa* had a 10 mm inhibition zone against *S.* *enterica* Typhi. On the other hand, in the study conducted by Islam et al. (2010), the methanolic extract of *Curcuma zedoaria* (Family: *Zingiberaceae*) did not exhibit a significant antibacterial effect against *S*. Paratyphi and *S*. Typhi by forming an inhibition zone of 8–9 mm.

Curcumin has been shown, in earlier research, to have broad-spectrum inhibitory effects on both gram-positive and gram-negative bacteria (Tyagi et al. 2015; Adamczak et al. 2020). In a way that supports this, our study observed that 200 mg/ml of curcumin had an antibacterial effect on *S*. Typhimurium by forming a zone diameter of 13 mm. Curcumin provides this antibacterial activity by inhibiting the bacterial quorum sensing system (QS) and by preventing the bacteria from adhering to host receptors. Also, curcumin demonstrates antibacterial activity by inhibiting the development and division of cells, the creation of biofilms, and the mobility of bacteria (Dai et al. 2022). On the other hand, *S*. Typhimurium is an important foodborne pathogen that can move with its flagellum. These flagella, which have an essential virulence feature, cross the intestinal barrier and cause disease. Studies have reported that curcumin slows down the motility of *S*. Typhimurium (Marathe et al. 2016). This hypothesis was supported in our research. This hypothesis was supported by curcumin’s antibacterial efficacy against *S*. Typhimurium in the current investigation.

### Microbiological analyses

Poultry meat and products have been recognised worldwide as a primary source of foodborne pathogens, such as *Salmonella* and *Campylobacter* spp. (Wang et al. 2023). According to the Regulation on Microbiological Criteria in Europe and Türkiye, *Salmonella* spp. must not exist in 25 g samples (Commission Regulation) (EC 2005). The number of *S.* Typhimurium at 0, 2, 4, and 6 days of storage were examined in the present study after experimental contamination of the chicken meat with *S.* Typhimurium at the level of 2.8 × 10^–7^ CFU/ml and then application of 1, 2 and 3% curcumin.

The solvents used may affect the antimicrobial tests. As seen in Table 1, the number of bacteria decreased by 1.89 logs in the control group containing the solvent DMSO only (no curcumin). In the 1% curcumin (containing DMSO) group, the bacteria count decreased by 1.90 logs. In the 3% curcumin group, the bacteria count decreased by 2.67 logs. It was observed that the effectiveness of the solvent (DMSO) alone was low, but its efficacy increased when used with curcumin. At the end of the 6^th^ day of storage, the number of *S.* Typhimurium did not differ significantly compared to the control group, but the number of bacteria in the 1, 2 and 3% curcumin applied groups decreased by 2.37, 2.71, and 2.84 logs.

Similar to the findings of the present study, in a study conducted by Altunatmaz et al. (2016), minced meat samples were contaminated experimentally with *S.* Typhimurium at a level of 10^–4^ CFU/g and then treated with 0.5, 1 and 2% curcumin. On the seventh day of storage, the number of *S.* Typhimurium decreased by approximately 2 logs in the group that was treated with 1% and 2% curcumin, but a decrease of 1 log was detected in the group that was treated with 0.5% curcumin. Previous studies show that antibacterial activity increases in direct proportion to the dose of curcumin.

The antimicrobial properties of curcumin (CUR) have been evaluated for efficacy in different media and various food matrices. Some researchers have shown that using curcumin and a photodynamic therapy is effective in reducing *Salmonella*. Gao and Matthews (2020) investigated the activity of water-soluble photosensitive curcumin (PSC) in inactivating *Salmonella* on the media and chicken skin. When *Salmonella* was treated with 200 ppm of CUR with 10 min of exposure to light on a media, the bacteria count decreased by 1.8 to 3.6 log10 CFU/ml. The *Salmonella* was reduced by approximately 1.5 log10 CFU/cm^2^ at 300 ppm PSC after a 5-minute incubation period and 32.1 kJ/m^2^ illumination on the chicken skin.

In another investigation, Gao et al. (2023) infected chicken skin with *Salmonella* and then treated it with 300 ppm of CUR for 5 min with light activation (430 nm, 5 minutes). After treating the chicken skin with photosensitiser curcumin (PSC), the *Salmonella* was reduced by 1.5 log CFU/cm^2^. The efficacy of PSC in controlling *Salmonella* was found to be equivalent to or better than the treatment using 300 ppm of peracetic acid (PAA).

In the study conducted by Urrutia et al. (2023), the *in vitro* antimicrobial activity of the photoactive compound curcumin (CUR) after light exposure of 430 nm for 0–5 min was investigated. It was found that when *S*. Typhimurium was treated with CUR at 100, 500, or 1 000 mg/l, the bacteria count was reduced between 4.73 and 4.90 log10 CFU/ml. It was stated that no discernible decrease was detected in the *Salmonella* after being treated with CUR. However, the number of *Salmonella* was found below detectable limits (5 CFU/ml) after being treated with 300 ppm of PAA. Our study, as in previous studies, showed that curcumin alone can inhibit *S*. Typhimurium without light exposure.

Different studies have been conducted to increase the effectiveness of curcumin in antimicrobial foods. Wang et al. (2024) designed a super absorbent, resilient antibacterial aerogel with curcumin for fresh pork preservation. They stated that *S. *Typhimurium significantly decreased to less than 45% with an increase in the CUR content. They reported that pork meat treated with an antimicrobial airgel pad (G3D1BC0.5-Cur0.7) effectively reduces the bacterial growth and can extend the freshness period by at least 12 days.

On the other hand, Zermeno-Ruiz et al. (2022) examined the effects of curcumin on *S.* Typhimurium at a level of 10^–7^ CFU/ml *in vitro* spectrophotometrically. Contrary to our study findings, they concluded that curcumin did not inhibit the bacterial growth after 2 h at 110, 220, and 330 μg/ml concentrations.

### Sensory evaluation

Curcumin, obtained from the *Curcuma longa *L. plant, has a distinctive slightly sharp odour and a bitter taste. It is used as a food additive with the code E 100 in food preservation as a preservative, colourant, and sweetener (Priyadarsini et al. 2003; Adamczak et al. 2020).

The colour of meat is an important physical parameter that affects consumers’ preferences. The colour of meat products depends on various factors, such as the composition of the raw material, the additives added to the product, and the cooking temperature (Kilic et al. 2021).

Since curcumin has a dominant yellow colour, it is preferrable to use it in low doses in foods. As the dose increases, the acceptability of the food in terms of the colour and odour decreases. In the present study, sensory changes in chicken meats treated with 1, 2, and 3% of curcumin were evaluated in terms of the colour, taste, odour, structure, and appearance. In this respect, chicken meat treated with 1% and 2% curcumin was accepted in terms of the colour, odour, taste, and appearance, while the 3% curcumin was found unacceptable.

Similar to the results of previous studies, it was found that as the dose of curcumin increased, an undesirable colour formed, though its antimicrobial characteristics increased, its sensory characteristics decreased (Altunalmaz et al. 2016). Like our study results, in the study conducted by Sujarwanta et al. (2019), it was found that the fortification of curcuma (*Curcuma zanthorrhiza*) flour by sensory tests showed no effect on the acceptability and that chicken nuggets with 2.0% turmeric flour showed the best chicken nugget characteristics.

In this study, it was revealed that the application of 1, 2, and 3% curcumin to chicken samples decreased the number of *S.* Typhimurium by 2.37, 2.71, and 2.84 logs at the end of the 6^th^ day. The highest decrease was detected with 3% curcumin by approximately 3 logs. As the dose of curcumin increased, the acceptability of the organoleptic characteristics of the chicken meat decreased, and the acceptable dose of curcumin was found to be appropriate up to 2%.

In the present study, it is suggested that curcumin, which is a natural agent obtained from the rhizome of the turmeric plant, can be used in foods for antibacterial purposes and to extend the shelf life.
